# Functional polymorphisms and transcriptional analysis in the 5′ region of the human serotonin receptor 1B gene (*HTR1B*) and their associations with psychiatric disorders

**DOI:** 10.1186/s12888-020-02906-4

**Published:** 2020-10-09

**Authors:** Xi Xia, Mei Ding, Jin-feng Xuan, Jia-xin Xing, Jun Yao, Xue Wu, Bao-jie Wang

**Affiliations:** School of Forensic Medicine, China Medical University, No. 77 Puhe Road, Shenbei New District, Shenyang, 110122 China

**Keywords:** *HTR1B*, Polymorphism, Transcriptional regulation, Schizophrenia, Psychiatric disorder

## Abstract

**Background:**

The 5-hydroxytryptamine 1B receptor (5-HT1B) plays an essential role in the serotonin (5-HT) system and is widely involved in a variety of brain activities. *HTR1B* is the gene encoding 5-HT1B. Genome-wide association studies have shown that *HTR1B* polymorphisms are closely related to multiple mental and behavioral disorders; however, the functional mechanisms underlying these associations are unknown. This study investigated the effect of several *HTR1B* haplotypes on regulation of gene expression in vitro and the functional sequences in the 5′ regulatory region of *HTR1B* to determine their potential association with mental and behavioral disorders.

**Methods:**

Six haplotypes consisting of rs4140535, rs1778258, rs17273700, rs1228814, rs11568817, and rs130058 and several truncated fragments of the 5′ regulatory region of *HTR1B* were transfected into SK-N-SH and HEK-293 cells. The relative fluorescence intensities of the different haplotypes and truncated fragments were detected using a dual-luciferase reporter assay system.

**Results:**

Compared to the major haplotype T-G-T-C-T-A, the relative fluorescence intensities of haplotypes C-A-T-C-T-A, C-G-T-C-T-A, C-G-C-A-G-T, and C-G-T-A-T-A were significantly lower, and that of haplotype C-G-C-A-G-A was significantly higher. Furthermore, the effects of the rs4140535T allele, the rs17273700C-rs11568817G linkage combination, and the rs1228814A allele made their relative fluorescence intensities significantly higher than their counterparts at each locus. Conversely, the rs1778258A and rs130058T alleles decreased the relative fluorescence intensities. In addition, we found that regions from − 1587 to − 1371 bp (TSS, + 1), − 1149 to − 894 bp, − 39 to + 130 bp, + 130 to + 341 bp, and + 341 to + 505 bp upregulated gene expression. In contrast, regions − 603 to − 316 bp and + 130 to + 341 bp downregulated gene expression. Region + 341 to + 505 bp played a decisive role in gene transcription.

**Conclusions:**

*HTR1B* 5′ regulatory region polymorphisms have regulatory effects on gene expression and potential correlate with several pathology and physiology conditions. This study suggests that a crucial sequence for transcription is located in region + 341 ~ + 505 bp. Regions − 1587 to − 1371 bp, − 1149 to − 894 bp, − 603 to − 316 bp, − 39 to + 130 bp, and + 130 to + 341 bp contain functional sequences that can promote or suppress the *HTR1B* gene expression.

## Background

Serotonin or 5-hydroxytryptamine (5-HT) is a crucial neurotransmitter that is widely involved in various physiological activities (e.g., mood, sleep, pain, feeding, and temperature regulation) [1]. A variety of mental or behavioral disorders are associated with 5-HT system dysfunction. The 5-HT receptor family includes seven categories and 14 subtypes [2]. The 5-HT 1B receptor (5-HT1B) is encoded by *HTR1B* and is closely associated with a variety of mental illnesses [3–6]. In previous behavioral studies, *HTR1B* knockout mice showed increased impulsivity and aggressiveness in certain behavioral phenotypes compared to wild-type mice [7]. Doxycycline restored *HTR1B* expression in adult mice and reversed the impulsive phenotype but not the aggression. *HTR1B* is located on chromosome 6, within 6q13-q26. Evidence suggests that there is a susceptibility gene for schizophrenia in this area [8]*.* HTR1B is also thought to be involved in the pathogenesis of major depression and antidepressant treatment. Selective ablation of 5-HT1B in mice caused increased extracellular serotonin levels following the administration of a selective serotonin reuptake inhibitor (SSRI) and induced antidepressant-like effects [9].

Many genetic polymorphisms are located in the *HTR1B* coding region and 5′ and 3′ regulatory regions. Genome-wide association studies (GWAS) have mapped thousands of variations associated with diseases. Over 90% of the variants are located in non-coding regions of the genome and do not directly affect the protein function. Thus variations in the non-coding sequences may be more likely to cause disease [10]. Duan et al. demonstrated that polymorphisms in the 5′-untranslated region of the *HTR1B* gene affect gene expression. T-261G mutation generates a new AP2 binding site that enhances transcriptional activity while the A-161 T mutation results in reduced transcriptional activity because of its different binding characteristics for AP1 [8]. The rs9361233 and rs9361235 polymorphisms are located in the transcription factor binding sites (TFBSs) in *HTR1B* and are significantly associated with clinical improvement after treatment with fluoxetine [11]. rs13212041 is located in the 3′ regulatory region of *HTR1B*. The rs13212041 G allele attenuates microRNA-directed mRNA silencing of *HTR1B* [12]. Individuals with the rs13212041 AA homozygous genotype are more aggressive than those that are G allele carriers. Conner et al. found that males with a low expression haplotype, who carry the rs13212041 A allele tend to exhibit greater anger and hostility [13]. Polymorphisms also perform functional effects in the form of haplotypes, and interaction between polymorphic genes also influence susceptibility and outcome of common complex diseases, such as mental illnesses [14]. The A-A-A-C haplotype of the rs6297, rs130058, rs1213366, and rs1213371 polymorphisms in the promoter region of *HTR1B* have a potential association with the gender of schizophrenic patients in the Spanish population [15]. Carriers of rs6296 and rs6298 G-T haplotype were estimated to have a higher risk of suicide attempts, and haplotype C-T was associated with a higher risk of depression in the younger age [16]. In a previous study, our group found that in the northern Han Chinese population, there were at least six SNPs in the 5′ regulatory region of *HTR1B*, including rs4140535, rs1778258, rs17273700, rs1228814, rs11568817, and rs130058. All these SNPs were located in the promoter region and the 5′ untranslated region (5′ UTR) and had minimum allele frequencies of more than 0.01. Among these SNPs, except for rs1228814 and rs17273700 that have not been reported to be associated with any physical disorders, all other SNPs have been found to be related to certain diseases. rs1778258 appears to be associated with schizophrenia. Other studies showed that rs4140535 could be a genetic factor for obesity [17], and rs11568817 and rs130058 could be associated with substance abuse [18]. However, the mechanisms underlying these associations were not adequately clarified. Functional SNPs can regulate gene expression through the effect on transcription factor binding [19]. Thus, the effects of polymorphisms and potential regulatory sequences of *HTR1B* will be further investigated in this study. Then we propose the following hypothesis. The SNPs located in the regulatory region affects gene regulation by changing the affinity of the transcription factor to the genome sequences, thereby enhancing or inhibiting the gene transcription, which ultimately leads to changes in the expression of the receptor, so polymorphisms in the regulatory region of the *HTR1B* gene may be the cause of individual differences in the susceptibility of some complex diseases.

## Methods

### Ethical compliance

The study was approved by the China Medical University Review Committee. All samples were collected in accordance with the principle of informed consent.

### Samples

Based on the data of our previous study, we selected genomic DNA samples containing six haplotypes (rs4140535, rs1778258, rs17273700, rs1228814, rs11568817, and rs130058) to construct the pGL3 recombinants. (Table 1). The details of the SNPs were presented as follows (Table 2). The target gene was obtained from GenBank using the reference sequence NC_000006.12 (*Homo sapiens* chromosome 6, GRCh38.p7 Primary Assembly).

**Table 1:** The polymorphism comparison of six SNPs in the haplotype recombinants

Haplotype	rs4140535	rs1778258	rs17273700	rs1228814	rs11568817	rs130058
H1	T	G	T	C	T	A
H2	C	A	T	C	T	A
H3	C	G	T	C	T	A
H4	C	G	C	A	G	T
H5	C	G	T	A	T	A
H6	C	G	C	A	G	A

**Table 2:** The information of the studied SNPs

SNP	Chr. pos	Base change	MAF	REGION
rs4140535	77,465,335	T > C	0.375	5’near gene
rs1778258	77,464,492	G > A	0.113	5’near gene
rs17273700	77,464,263	T > C	0.121	5’near gene
rs1228814	77,464,103	C > A	0.166	5’near gene
rs11568817	77,463,665	T > G	0.116	5’UTR
rs130058	77,463,564	A > T	0.077	5’UTR

*MAF* Minimum allele frequency

### Construction of haplotype recombinants in the 5′ regulatory region of *HTR1B*

According to data from DNA sequencing in our previous study, DNA samples containing haplotypes H1 to H6 were used as DNA templates for PCR. The target fragments spanned from −1587 to 711 bp in the 5′ regulatory region (TSS, + 1). The 5′ end of the primers introduced KpnI and XhoI cleavage sites. The primer sequences were 5′-GGGGTACCCGGTTTGTGCTTTATTGCCTT-3′ (sense), and 5′-CCGCTCGAGCAGAGGATAAGTTGGCTTG-3′ (antisense). The target fragments were cloned into the pBM20S vector (Biomed, Beijing, China), and then re-cloned into the KpnI/XhoI site of the promoterless and enhancerless reporter vector pGL3-Basic (Promega, Madison, WI). The target haplotype of each construct was verified by DNA sequencing.

### Construction of truncated sequence recombinants in the 5′ regulatory region of *HTR1B*

**Fig. 1:**
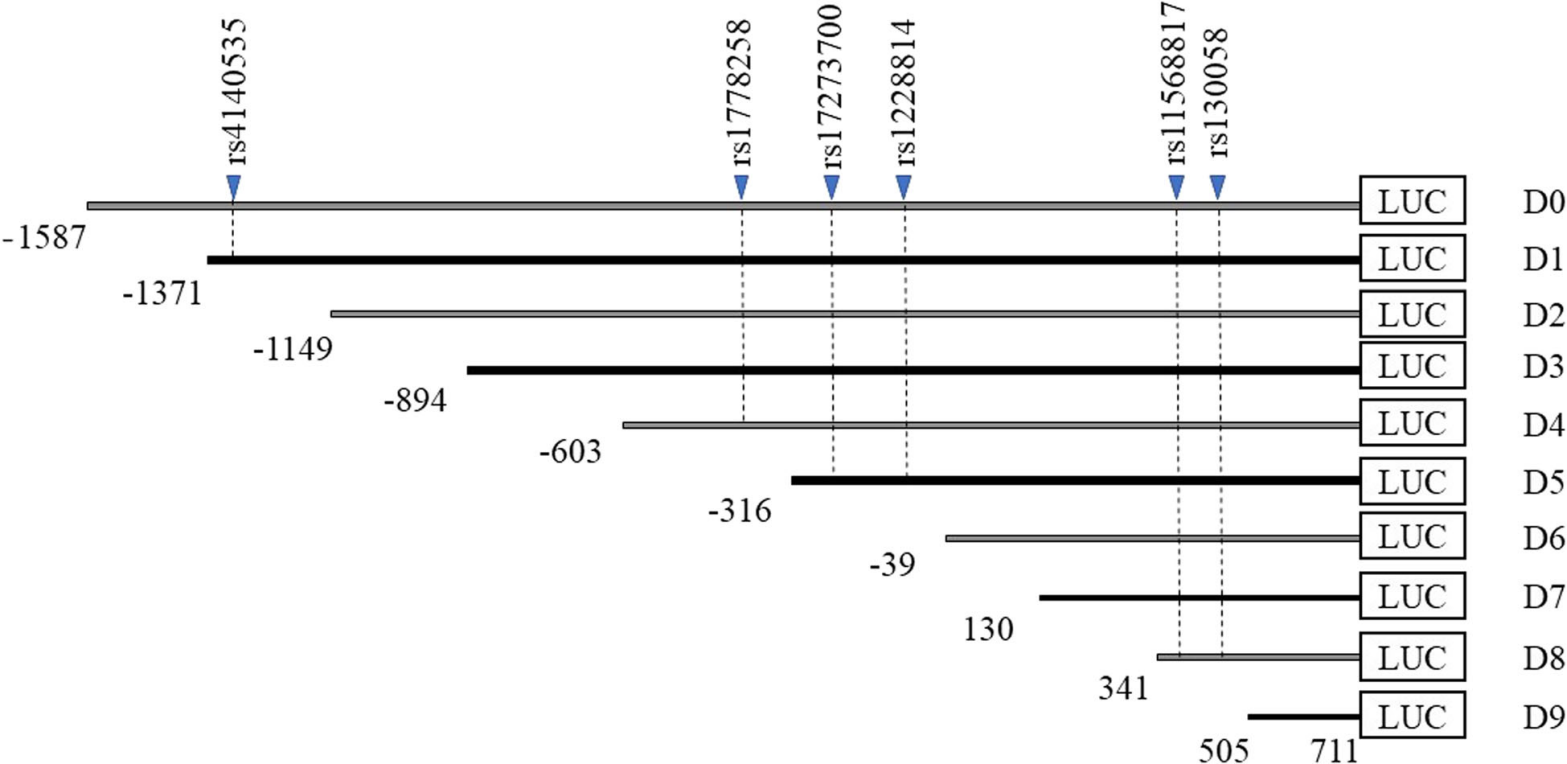
Ten truncated sequence recombinants in the 5′ regulatory region of *HTR1B*. The target gene was obtained using the GenBank reference sequence NC_000006.12 (*Homo sapiens* chromosome 6, GRCh38.p7 Primary Assembly). The ten DNA fragments shared a common 3′ end at + 711 bp (TSS, + 1). The longest fragment was from − 1587 to + 711 bp, and the shortest fragment was from + 505 to + 711 bp. The SNPs overlap with some of the truncated regions

The amplified targets consisted of ten DNA fragments with a common end at + 711 bp (TSS, + 1). The longest fragment was from − 1587 to + 711 bp, and fragments of different lengths were sequentially truncated. The shortest fragment was from + 505 to + 711 bp (Fig. 1). Ten pairs of primers were used for PCR amplification of the target fragments (Table 3). KpnI and XhoI cleavage sites were introduced into the 5′ end of the primers. The target gene was cloned into the KpnI/XhoI site of pGL3-Basic in the same manner as for the haplotype recombinants. The truncated sequence recombinants were verified by DNA sequencing.

**Table 3:** Primer sequences of the target fragments

Primer	sequences
D0 (− 1587 ~ + 711)-F	5′ GGGGTACCCGGTTTGTGCTTTATTGCCTT 3’
D1 (− 1371 ~ + 711)-F	5′ GGGGTACCAGGAATTCGTAGTGGGGATCAT 3’
D2 (− 1149 ~ + 711)-F	5′ GGGGTACCGCAAAGGAGACACACAAGATG 3’
D3 (− 894 ~ + 711)-F	5′ GGGGTACCTGTCTTTTTCCCTGTTGACTCATAG 3’
D4 (−603 ~ + 711)-F	5′ GGGGTACCTAAGGACATGGGTCTCACTGG 3’
D5 (−316 ~ + 711)-F	5′ GGGGTACCGGACCGGACCTGGACTCTATAT 3’
D6 (−39 ~ + 711)-F	5′ GGGGTACCGTGGCCAGAGAGTGAAAAGAG 3’
D7 (+ 130 ~ + 711)-F	5′ GGGGTACCCTGCAAGCTTTGGTCTCTACAC 3’
D8 (+ 341 ~ + 711)-F	5′ GGGGTACCAACCCAGGTCTAAGACCCGGT 3’
D9 (+ 505 ~ + 711)-F	5′ GGGGTACCGAACTATCAACTGGGGACAAAC 3’
Common end (+ 711)-R	5′ CCGCTCGAGCAGAGGATAAGTTGGCTTG 3’

### Cell lines and cell culture

The human neuroblastoma (SK-N-SH) and embryonic kidney (HEK-293) cell lines were obtained from the Cell Bank of the Chinese Academy of Sciences (Shanghai, China) [20, 21]. SK-N-SH and HEK-293 cells were cultured in HyClone® DMEM supplemented with 15% FBS (PAN-Biotech) and KeyGEN BioTECH® DMEM in the presence of 10% FBS, respectively. All cells were grown in a humidified 37 °C environment at 5% CO_2_.

### Transfection and dual-luciferase reporter assay

Cells were plated in 24-well plates (2 × 10^5^ cells/well) and grown for 36 to 48 h to 70 to 90% confluence. The pGL3 recombinants (500 ng) or control plasmid pGL3-Control (Promega, Madison, WI) were transiently transfected using Lipofectamine®3000 reagent (Invitrogen, CA), according to the manufacturer’s protocol. pRL-TK (50 ng) (Promega, Madison, WI) was co-transfected with each plasmid to normalize for transfection efficiency. Cell lysates were collected for the reporter assay 28 to 30 h post-transfection. The reporter assay was carried out using the Dual-Luciferase® Reporter Assay System (Promega, Madison, WI). The firefly luciferase activity (LUC) was normalized to the renilla luciferase activity (TK). Each recombinant was tested in triplicate per experiment with total of three experiments.

### Statistical analysis

The relative fluorescence intensity was calculated by dividing the LUC value by that of TK [22]. The comparison between the different recombinants was performed using SPSS19.0 software (IBM, Armonk, NY, USA). All groups of data were verified to follow a normal distribution. Differences in relative fluorescence intensity between each two haplotype recombinants were calculated using Dunnett’s T3 test as a post hoc test following one-way analysis of variance (ANOVA). Differences in the relative fluorescence intensities between adjacent truncated sequence recombinants (D0 versus D1, D1 versus D2, D2 versus D3, D3 versus D4, D4 versus D5, D5 versus D6, D6 versus D7, D7 versus D8, and D8 versus D9) were calculated using the independent-samples *t*-test. *P* < 0.05 indicated statistical significance.

### Bioinformatics platform prediction

HaploReg v4.1 and regulome DB was used to detect the information on the SNPs in regulatory function. Transcription factor predictions for the functional fragments were carried out using JASPAR (http://jaspar.genereg.net) [23]. The filter was set to a threshold of 85%.

## Results

### The polymorphisms of the 5′ regulatory region of *HTR1B* have regulatory effects on transcriptional activity

**Fig. 2:**
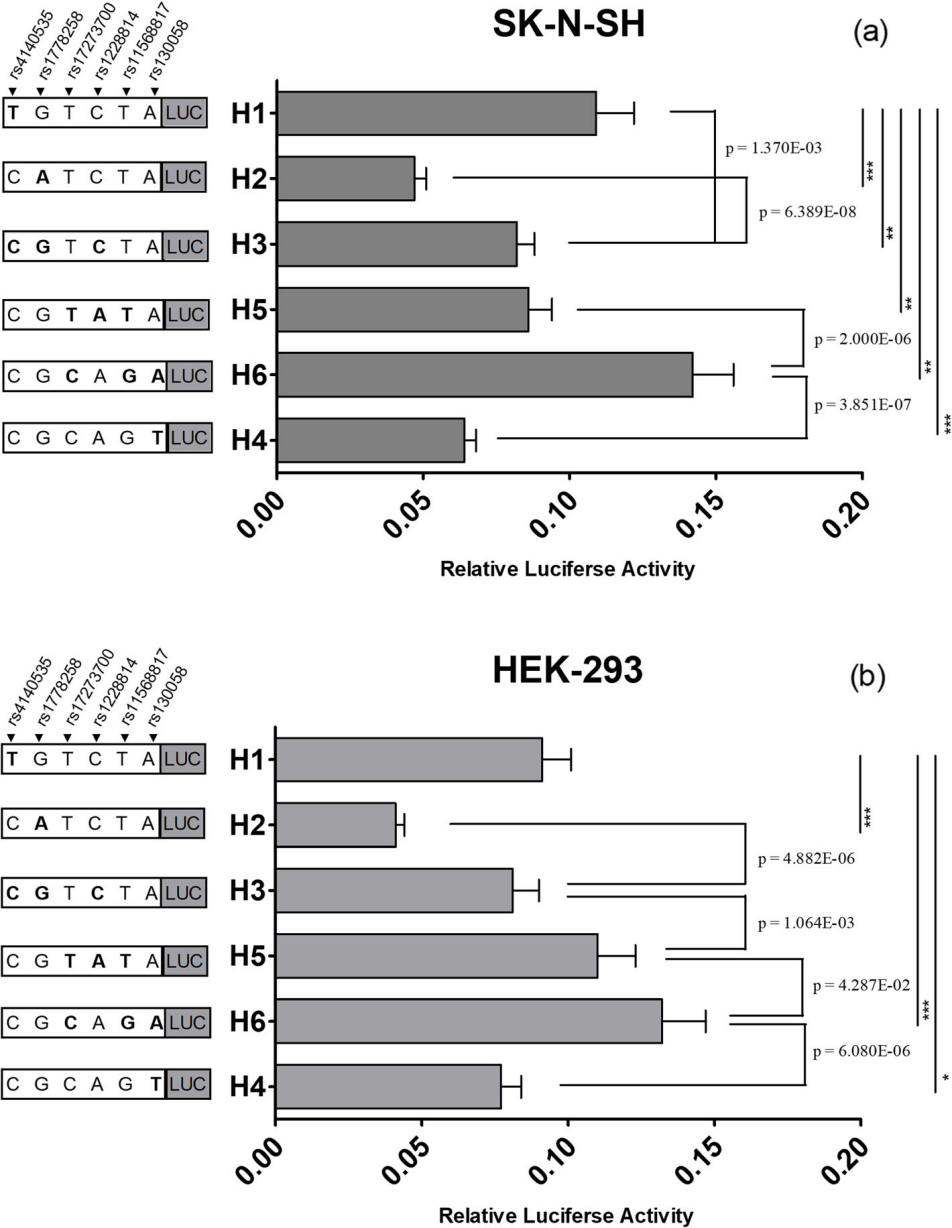
Relative fluorescence intensities of the haplotype recombinants in SK-N-SH and HEK-293 cells. The haplotypes H2, H3, H4, H5, and H6 showed significantly different luciferase activity compared to the major haplotype H1 in SK-N-SH cell (**a**), the haplotype H2, H4 and H6 also showed significantly different luciferase activity compared to the major haplotype H1 in HEK-293 cell (**b**). ***p < 0.001; **p < 0.01, *p < 0.05. The relative fluorescence intensity of haplotype H1(with rs4140535T allele) was significantly higher than that of haplotype H3 (with rs4140535C allele) in the SK-N-SH cells, The relative fluorescence intensity of haplotype H2 (with rs1778258A allele) was much lower than that of haplotype H3 (with rs1778258G allele) in both cell lines, The haplotype H5 (with rs1228814A allele) appeared to upregulate the luciferase activity relative to haplotype H3 (with rs1228814C) in HEK-293 cells, the haplotype H6 (with rs17273700C-rs11568817G) had higher relative luciferase intensity than haplotype H5 (with rs17273700T-rs11568817T), and haplotype H4 (with rs130058T allele) had lower relative luciferase intensity than haplotype H6 (with rs130058A allele) in both SK-N-SH and HEK-293 cell lines (**a,b**)

In the previous study, we found that haplotype H1 was the major haplotype in the northern Han Chinese population. We used a one-way analysis of variance to identify the differences in relative fluorescence intensity between each two haplotype recombinants in SK-N-SH and HEK-293 cell lines (*p* = 2.2489E-26, *p* = 2.1152E-22). Based on the results of Levene’s test (*p* = 0.026, *p* = 0.004), Dunnett’s T3test was used as a post hoc test. In SK-N-SH cells, the less prominent haplotypes H2, H3, H4, and H5 had significantly lower relative fluorescence intensities compared to H1 (*p* = 1.892E-06, 1.370E-03, 2.337E-05, and 6.123E-03, respectively). In contrast, haplotype H6 demonstrated increased transcriptional activity compared to H1 (*p* = 1.266E-03) (Fig. 2a). Haplotypes H2, H4, and H6 caused similar effects in HEK-293 cells (*p* = 1.986E-06, 3.977E-02, and 1.017E-04, respectively) (Fig. 2b).

We observed a variation between haplotypes H1 and H3 in rs4140535 (T/C). The relative fluorescence intensity of haplotype H1 was significantly higher than that of haplotype H3 in the SK-N-SH cells, indicating that the rs4140535T allele increased *HTR1B* gene expression. We also noticed that there was a difference between haplotypes H2 and H3 at rs1778258 (A/G). The relative fluorescence intensity of haplotype H2 was much lower than that of haplotype H3 in both cell lines, suggesting that the rs1778258A allele had a negative effect on *HTR1B* transcriptional activity. The rs1228814A allele of H5 also appeared to upregulate *HTR1B* gene expression relative to haplotype H3 in HEK-293 cells. Haplotype H6 had the highest relative fluorescence intensity. The variations between haplotypes H5 and H6 were present in rs17273700 (T/C) and rs11568817 (T/G), and there was a strong linkage between rs17273700 and rs11568817 (T-T/C-G). Thus, the C-G combination enhanced *HTR1B* gene expression. Haplotypes H4 and H6 exhibited different transcriptional effects in the two cell lines. Based on their variation at rs130058 (A/T), the rs130058T allele may inhibit *HTR1B* gene expression.

### Analysis of functional sequences of 5′ regulatory region of *HTR1B*

**Fig. 3:**
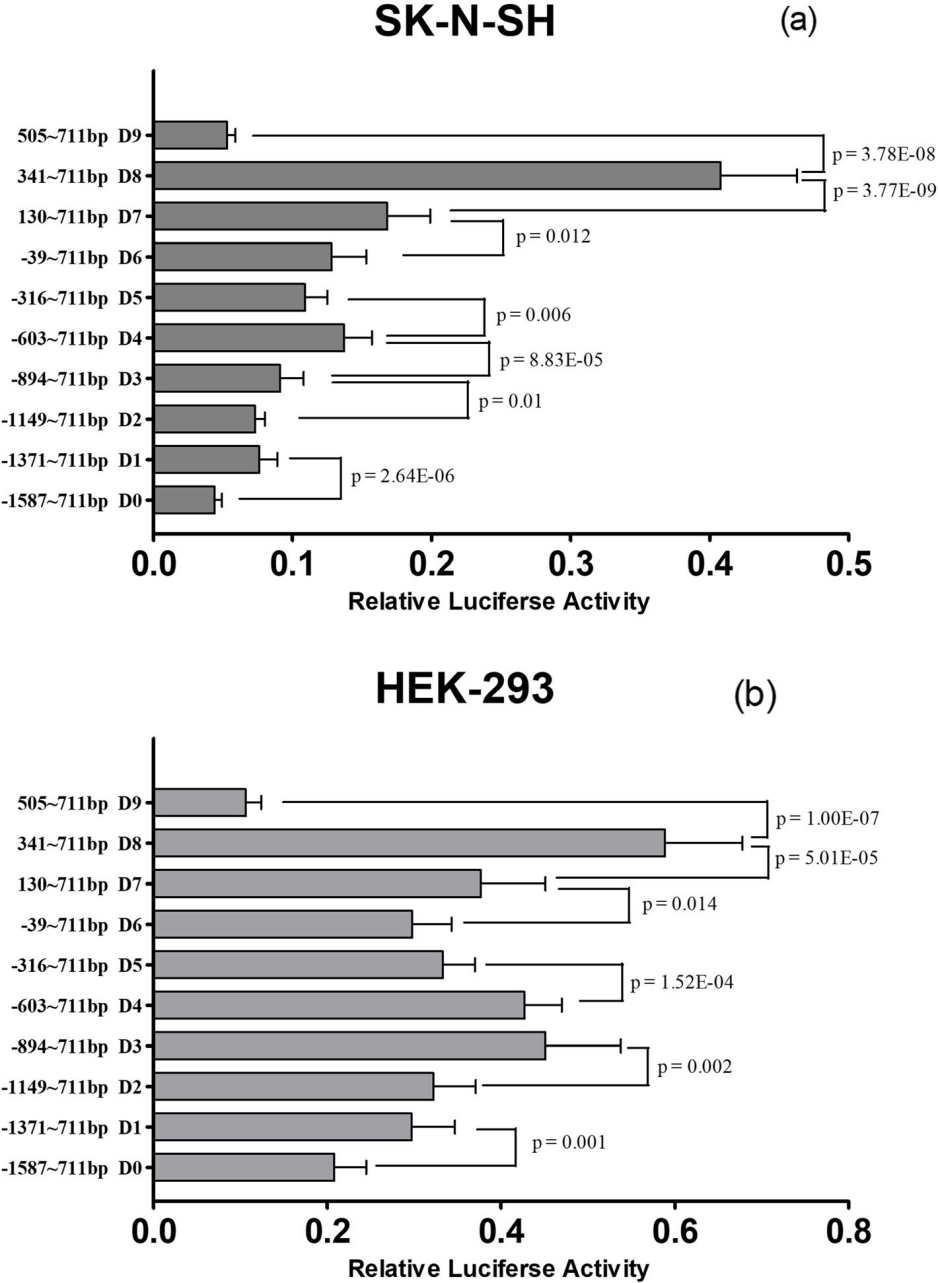
Relative fluorescence intensities for ten truncated sequence recombinants in SK-N-SH and HEK-293 cells. There was a significant difference in the relative fluorescence intensities between D0 versus D1, D2 versus D3, D2 versus D3, D3 versus D4 (SK-N-SH only), D4 versus D5, D6, and D7, D7 versus D8, and D8 versus D9. The data are presented as the mean ± SD

In SK-N-SH, the changes in the relative fluorescence intensities between the truncated fragments D0 and D1, D2 and D3, D3 and D4, D6 and D7, and D7 and D8 showed an upward trend, while the differences between D4 and D5 and D8 and D9 fragments exhibited a downward trend (Fig. 3). The trends were similar in both cell lines, except for the comparison of D3 and D4, where a significant difference was only observed in SK-N-SH cells. Overall, our results suggested that the sequences between − 1587 and − 1371 bp (D0-Dl), − 1149 and − 894 bp (D2-D3), − 39 and + 130 bp (D6-D7), and + 130 and + 341 bp (D7-D8) had negative effects on *HTR1B* gene expression while the sequences located between − 603 and − 316 bp (D4-D5) and + 341 and 505 bp (D8-D9) promoted *HTR1B* gene expression.

### Results of bioinformatics platform prediction

**Fig. 4:**
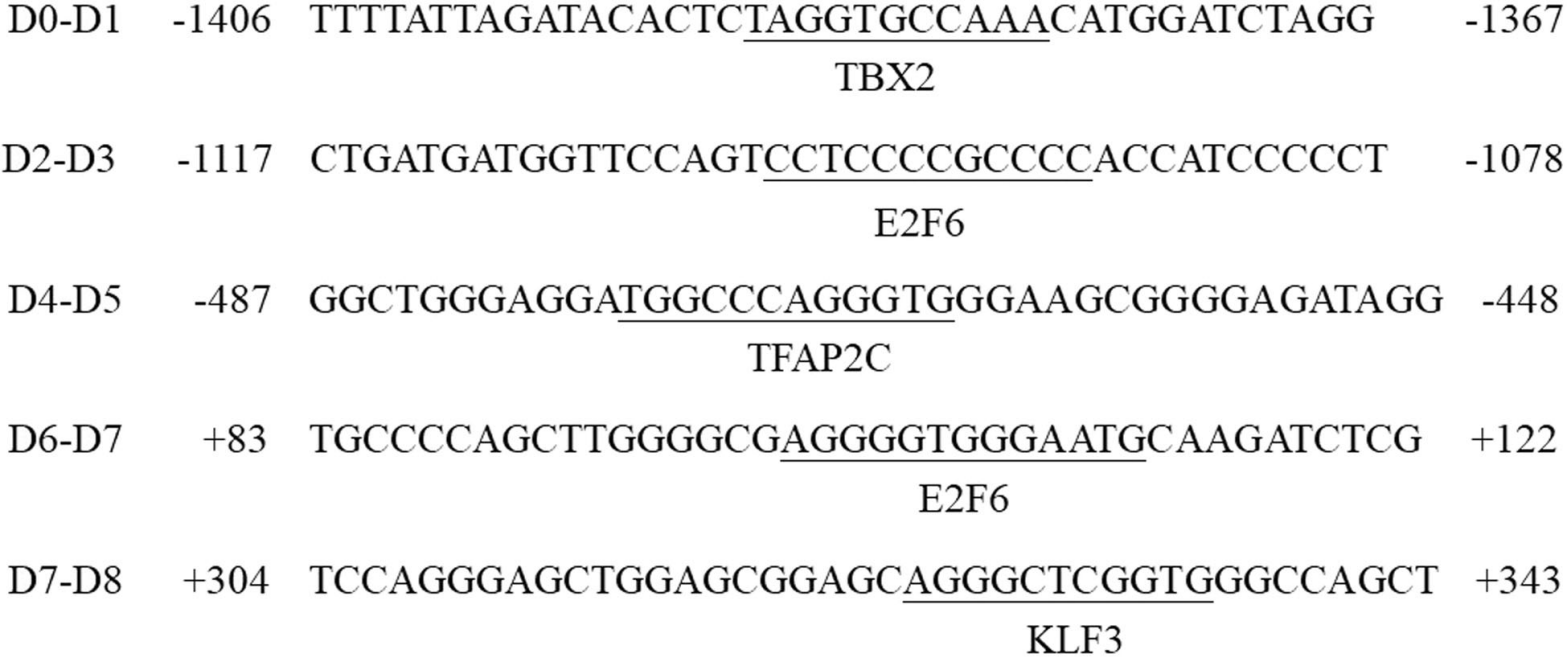
The prediction of transcription factor. The predictive results for transcription factor binding in the functional fragments are shown. The numbers in the figure annotate the position of the fragment (TSS, + 1)

We obtained the information for studied SNPs in HaploReg and Regulome DB. Rs4140535, rs1778258, rs17273700, rs1228814, rs11568817, and rs130058 were showed overlap in regions with promoter or enhancer regions (Table 4). We used JASPAR to predict the transcription factors of the functional fragments, which are − 1587 ~ − 1371 bp (D0-Dl), − 1149 ~ − 894 bp (D2-D3), − 603 ~ − 316 bp (D4-D5), − 39 ~ + 130 bp (D6-D7), and + 130 ~ + 341 bp (D7-D8). Combined with statistical scores and expression in tissues, especially brain tissues (HPA RNA-seq normal tissues project), and their role in the central nervous system, we screened for the most relevant transcription factors (Fig. 4).

**Table 4:** Information for studied SNPs in HaploReg and Regulome DB

Variant	Promoter/enhancer histone marks	Regulatory motifs altered
rs4140535	12_EnhBiv	5 altered motifs BATF_disc3, GATA_disc3, STAT_known11 etc.
rs1778258	12_EnhBiv, 2_PromU,23_PromBiv	CTCF,Rad21,TR4
rs17273700	12_EnhBiv, 2_PromU,23_PromBiv	7 altered motifs Ahr::Arnt::HIF, BHLHE40_disc2, HEY1_disc2 etc.
rs1228814	12_EnhBiv, 2_PromU,23_PromBiv	8 altered motifs CCNT2_disc2, Ets_disc1, PLAG1 etc.
rs11568817	3_PromD1, 12_EnhBiv, 2_PromU,23_PromBiv	
rs130058	3_PromD1, 12_EnhBiv, 2_PromU,23_PromBiv	9 altered motifs GCNF, Nr2f2, RXRA_known1 etc.

## Discussion

Complex diseases such as cancer and psychiatric disorders are genetic diseases related to multiple genomic variants. Increasing studies have shown that compared with mutations in the coding regions, variations associated with the regulatory mechanisms are more likely to cause diseases related to complex traits [10]. The fine-tuning on the post-transcriptional level is important to gene regulation. The fate of mature mRNA is affected by non-coding RNAs and RNA-binding proteins (RBPs). RBPs, such as transcription factor, is a crucial regulator for post-transcriptional regulation, and the regulation of the RBPs is highly dependent on the binding sequences [24]. Information on the studied SNPs assessed in HaploReg and Regulome DB datasets showed that rs4140535, rs1778258, rs17273700, rs1228814, rs11568817, and rs130058 were all overlap in regions with promoter or enhancer function in tissues such as the brain. Among them, rs1778258 and rs17273700 were found to have bound proteins. Also, the studied SNPs except for rs11568817 were showed altered regulatory motifs at their positions. The previous study found that rs11568817 and rs130058 changed the transcription factor binding features, which is inconsistent with the datasets [8]. Thus, the function of these SNPs is still worthy of further investigation.

In this study, six haplotype recombinants were transfected into SK-N-SH and HEK-293. We observed that haplotypes H1 showed a higher fluorescence activity than haplotype H3 (SK-N-SH). Therefore, the rs4140535 C allele could inhibit transcriptional activity. Previous studies found that 5-HT1B plays an important role in regulating feeding behavior. It is suggested that rs4140535 could be a genetic factor for obesity in African Americans, and the rs4140535T allele was a protective allele against excessive BMI [17]. Blocking 5-HT1B eliminates the anorexia and weight loss caused by leptin and AM 251 [25], and serotonin-induced decreases in appetite require activation of 5-HT1B [26]. Our data suggest that the *HTR1B* polymorphism rs4140535T/C is likely related to weight gain.

Haplotype H3 was also observed to have a lower transcriptional activity than haplotype H5 (HEK-293). Thus, the rs1228814A allele of H5 appeared to upregulate *HTR1B* gene expression. Although there have been studies on the relationship between rs1228814 and depression and methamphetamine addiction [27, 28], there is currently no evidence associating rs1228814 with mental illness.

The H2 and H4 haplotypes were associated with decreased transcriptional activity, whereas haplotype H6 increased transcriptional activity in both cell lines. The relative fluorescence intensity of haplotypes H2 is lower than that of haplotypes H3. It shows that the rs1778258A allele has a negative regulatory effect on *HTR1B* gene expression. In our previous study, we found that the rs1778258A allele was potentially associated with schizophrenia in the northern Han Chinese population [29]. The function of rs1778258A/G was confirmed in the current study. Therefore, the association between rs1778258 and schizophrenia might be related to the effect of the rs1778258A/G polymorphism on gene expression. It should be noted that haplotype H2 caused the lowest *HTR1B* expression in both SK-N-SH and HEK-293. Although there was no evidence that this haplotype is associated with schizophrenia based on our previous studies, we cannot rule out that it may be related to other mental disorders caused by 5-HT1B dysfunction. Besides, the transcriptional effect of Haplotypes H4 was different from haplotype H6. Based on their variation at rs130058 (A/T), the rs130058T allele could significantly downregulate gene expression. rs130058 has been confirmed to bind to TFAP1, the rs130058A and rs130058T alleles exhibit different characteristics when binding to TFAP1, which affects the transcriptional activity of *HTR1B* [8]. In our previous study, we observed that the distribution of haplotype H6 was different between schizophrenic patients and healthy individuals in the northern Han Chinese population; however, the statistical difference was lost after the Bonferroni correction. In this study, haplotype H6 was found to significantly upregulated *HTR1B* gene expression, which could probably result in an increased level of 5-HT1B. However, there are no consistent findings in the relationship between the 5-HT1B receptor level and schizophrenia [30]. rs17273700 (T/C) and rs11568817 (T/G) variations were presented between haplotypes H5 and H6, and we have already confirmed a strong linkage between rs17273700 and rs11568817 (T-T/C-G) in the previous study [29]. We observed that the relative fluorescence intensity of the haplotype H6 was higher than that of haplotype H5. Thus, the C-G combination in rs17273700 and rs11568817 upregulated *HTR1B* expression. Duan et al. confirmed that the rs11568817G allele could generate a new TFAP2 binding site, resulting in improved transcriptional activity [8]. The TFAP2 family, which includes TFAP2A, TFAP2B, and TFAP2C, represents a group of transcriptional activators that regulate the expression of specific genes. Rs11568817 is also believed to be related to other psychiatric disorders, such as drug abuse, alcohol and nicotine dependence, and anxiety [18]. It is likely related to the sexual dimorphism of the serotoninergic system [31].

Not all of the specific binding sequences for transcription factors included polymorphisms. Ten different truncated fragments were transfected into SK-N-SH and HEK-293 cells. The trends of the changes in relative fluorescence intensity between D0 and D1, D2 and D3, D6 andD7, and D7 and D8 were upward, while the trends for changes between D4 and D5 and D8 and D9 were downward in both cell lines. Therefore, the deleted fragments ranging from − 1587 to − 1371 bp (D0-D1), − 1149 to − 894 bp (D2-D3), − 39 ~ + 130 bp (D6 ~ D7), and + 130 ~ + 341 bp (D7 ~ D8) contain transcriptional suppression regions, and − 603 ~ − 316 bp (D4-D5), + 341 ~ + 505 bp (D8-D9) contain enhancing regulatory regions for gene expression. The functional regions were analyzed by JASPAR (http://jaspar.genereg.net) [23], and we found many transcription factors that probably bind to *HTR1B*, such as TBX2, E2F6, TFAP2, and KLF3 (Fig. 4).

The bioinformatics platform predicted that the deleted fragment from − 1587 to − 1371 bp (D0-D1) contains the binding site for T-box transcription factor 2 (TBX2). This transcription factor is a transcriptional repressor of *ADAM10*. Its effects are mediated by binding to two TBX2 binding sites within the core promoter region of *ADAM10*, and substrate cleavage by ADAM10 has been implicated in pathological situations such as Alzheimer’s disease [32]. We also found that E2F6 binding sites were present in the deleted fragments from − 1149 to − 894 bp (D2-D3) and − 39 to + 130 bp (D6-D7). E2F6 plays a vital role in controlling the cell cycle and contains a modular suppression domain that has inhibitory effects on transcription [33, 34]. Our analysis also predicted that the deleted fragment from + 130 to + 341 bp (D7-D8) contains a binding site for Kruppel-like factor 3 (KLF3). KLF3 belongs to the family of Sp1/Kruppel-like zinc finger transcription factors. These transcription regulators modulate the expression of a large number of genes that have GC-rich promoters and may take part in all facets of cellular function [35]. KLF3 is widely expressed as a transcriptional repressor that functions by binding with the co-repressor protein C-terminal binding protein (CtBP) [36].

Deletion of the fragment from − 603 to − 316 bp (D4-D5) caused a decrease in transcription activity. This region contained a binding site for TFAP2C. Studies have shown that TFAP2C plays a role in cerebral cortex development, it directly regulates the basal progenitor fate determinants Tbr2 and NeuroD [37].. TFAP2C can also control hippocampal neurogenesis in adults and regulate cognitive behavior [38]. It was impressive that the luciferase activity of D8 was 7 to 8-fold higher than that of D9 in SK-N-SH cells. Therefore, the truncated fragment + 341 ~ + 505 bp (D8-D9) is likely to play a crucial role in gene transcription.

This study investigated the function of the 5′ regulatory region of *HTR1B.* However, limited by experimental conditions, it is unavailable for us to corroborate the results in the in vivo tissues. Besides, the mechanisms of the functional polymorphisms still require further exploration, and the potential functional regions must be verified with additional experiments. We attempt to perform animal experiments in the following studies to investigate the regulatory mechanism of the downstream pathways and mechanisms involving other variables such as alternative promoter or translational regulation, as well as the correlation between the function of *HTR1B* and psychiatric disorders.

## Conclusions

*HTR1B* 5′ regulatory region polymorphisms have regulatory effects on gene expression and may correlate with several pathological and physiological conditions, such as weight gain and schizophrenia. The results of this study suggest that the region from + 341 to + 505 bp (TSS + 1) is crucial for the transcription of the *HTR1B* gene. Regions − 1587 to − 1371 bp, − 1149 to − 894 bp, − 603 to − 316 bp, − 39 to + 130 bp, and + 130 to + 341 bp contain functional sequences that can promote or suppress *HTR1B* expression.

## Data Availability

All data generated or analyzed during this study are included in this published article.

## Abbreviations

HTR1B: Serotonin receptor 1B
5-HT: 5-hydroxytryptamine
5-HT1B: 5-hydroxytryptamine receptor 1B
SNP: Single-nucleotide polymorphism
UTR: Untranslated region
RBPs: RNA-binding proteins
TSS: Transcription start site
